# Conversations in the Gut: The Role of Quorum Sensing in Normobiosis

**DOI:** 10.3390/ijms24043722

**Published:** 2023-02-13

**Authors:** Victor Markus, Abraham Abbey Paul, Kerem Teralı, Nazmi Özer, Robert S. Marks, Karina Golberg, Ariel Kushmaro

**Affiliations:** 1 Department of Medical Biochemistry, Faculty of Medicine, Near East University, Nicosia 99138, Cyprus; 2 Avram and Stella Goldstein-Goren Department of Biotechnology Engineering, Ben-Gurion University of the Negev, Be’er Sheva 84105, Israel; 3 Department of Medical Biochemistry, Faculty of Medicine, Cyprus International University, Nicosia 99258, Cyprus; 4 Department of Biochemistry, Faculty of Pharmacy, Girne American University, Kyrenia 99428, Cyprus; 5 The Ilse Katz Center for Nanoscale Science and Technology, Ben-Gurion University of the Negev, Be’er Sheva 84105, Israel; 6 School of Sustainability and Climate Change, Ben-Gurion University of the Negev, Be’er Sheva 84105, Israel

**Keywords:** gut, bacteria, quorum sensing, autoinducers, normobiosis, dysbiosis, health

## Abstract

An imbalance in gut microbiota, termed dysbiosis, has been shown to affect host health. Several factors, including dietary changes, have been reported to cause dysbiosis with its associated pathologies that include inflammatory bowel disease, cancer, obesity, depression, and autism. We recently demonstrated the inhibitory effects of artificial sweeteners on bacterial quorum sensing (QS) and proposed that QS inhibition may be one mechanism behind such dysbiosis. QS is a complex network of cell–cell communication that is mediated by small diffusible molecules known as autoinducers (AIs). Using AIs, bacteria interact with one another and coordinate their gene expression based on their population density for the benefit of the whole community or one group over another. Bacteria that cannot synthesize their own AIs secretly “listen” to the signals produced by other bacteria, a phenomenon known as “eavesdropping”. AIs impact gut microbiota equilibrium by mediating intra- and interspecies interactions as well as interkingdom communication. In this review, we discuss the role of QS in normobiosis (the normal balance of bacteria in the gut) and how interference in QS causes gut microbial imbalance. First, we present a review of QS discovery and then highlight the various QS signaling molecules used by bacteria in the gut. We also explore strategies that promote gut bacterial activity via QS activation and provide prospects for the future.

## 1. Introduction

The gut ecosystem has the greatest concentration and variety of microbial species of any natural environment. An estimated 10^14^ bacteria and other microbial species including viruses, archaea, and fungi have been reported to inhabit the human gut [1]. This collection of microbial species coevolved with the host over time to create a delicate and beneficial association that essentially promotes health and well-being. It has been shown that the “gut microbiota “contains more bacterial cells—about 10 times as many as there are in other human organs—and its genomic content is more than 100 times as abundant as the human genome [2]. The major phyla of gut microbiota include Bacteroidetes, Firmicutes, Proteobacteria, Actinobacteria, Verrucomicrobia, and Fusobacteria, with Firmicutes and Bacteroidetes accounting for over 90% of the entire gut microbiota [3]. Evaluation of the makeup of microbial species found in the human gut reveals distinct inter-individual variances [4], with the average total number of diverse commensal bacterial strains approximated at over 500 per individual [5]. While the diversity of dominating species appears to be constant throughout time and between individuals, that of subdominant taxa is far less stable, largely as a result of genomic plasticity [4].

## 2. Normobiosis

The term “normobiosis” is used to describe the normal balance of the different types of microbial species in the gut. In a symbiotic association, the gut microbiota performs vital functions while the host offers a nutrient-rich environment [5]. The gut microbiota serves the host in a variety of physiological ways, including maintaining gut epithelium integrity, facilitating digestion, producing vital metabolites and vitamins, suppressing pathogen expansion, and regulating host immunity [1,3,6].

Despite the enormous variety of microorganisms and complexity observed in the adult intestine, the microbiota is initially an uncomplicated ecosystem that gradually goes through successional modifications until it reaches great diversity [7,8]. For many years, it was thought that the infant’s gut was sterile and that colonization after delivery came solely from the mother, nutrition, and the environment [9,10,11,12]. The discovery of bacterial DNA from placenta samples [13] and meconium [8] gave an indication initially that the placenta may be a possible pathway through which horizontal bacterial DNA transfer occurs from mother to fetus [13]. However, other investigations show bacteria in the umbilical cord blood [14] and meconium of healthy newborns including from murine amniotic fluid taken during a cesarean operation [15], thus indicating that bacterial exposure may begin in utero. Infants with a balanced gut microbiota have healthier growth and development, a stronger immune system, and a lower chance of developing chronic diseases [16]. Studies have indicated that the first bacteria to colonize the newborn gut are the facultative anaerobic bacteria such as *Staphylococcus*, *Streptococcus*, *Enterococcus*, and *Enterobacter* spp. [8,17]. By lowering the initial oxygen levels, the facultative bacteria open the way for anaerobes like *Bifidobacterium*, *Bacteroides*, and *Clostridium* spp. to begin colonizing the gut [8]. Several factors, like dietary changes and the use of antibiotics, have been shown to cause dysbiosis, an imbalance in gut microbiota. This dysbiosis is associated with a number of disorders such as inflammatory bowel disease, cancer, obesity, depression, and autism [18,19,20,21]. 

Normobiosis depends on several factors such as the mode of delivery, diet, host genotype, age, medication, and the environment in which an individual is born and raised (rural or urban).

### 2.1. Mode of Delivery

Mode of delivery has a significant impact on bacterial species that first colonize the infants’ gut [8]. Through vaginal deliveries, bacteria such as Bifidobacterial strains from the mother’s gut and vagina are passed to the infant’s gut [22,23,24], whereas cesarean deliveries introduce colonizing bacteria from the environment, such as from the hospital environment [25]. Examples of hospital bacteria include opportunistic pathogens like *Klebsiella* and *Enterococcus* [26].

### 2.2. Diet

The gut microbiota is highly dynamic during the first few weeks of life, with nutrients controlling the changing ecosystem [27]. Infants who are fed formula often have a microbiota that is more diverse than the microbiota of those who are breastfed, typically dominated by Bifidobacteria [8,28]. Following the introduction of solid foods, the bacterial succession continues to slowly diversify and includes adult-type species such as *Bacteroides* spp. and *Clostridium* clusters IV and XIV [8,17]. Although the precise age for the attainment of a permanent adult-type composition is still unknown, it is generally accepted that this age is in the range of 3 years [29,30,31]. Nonetheless, the process of gut microbiota modification continues after the age of 3, and later-life occurrences like hormonal changes associated with puberty or diet changes also have an impact on the composition of the microbiota [32,33]. Generally, a diet high in fiber and plant-based foods, and low in processed foods, is associated with a more diverse and balanced gut microbiota [34].

### 2.3. Genetics

Studies suggest that genetics can influence the composition of gut microbiota. One of the basic approaches to determining how host genotype influences gut microbiota is through twin studies [35]. Fraternal (dizygotic; DM) twins often share 50% of their genes compared to 100% shared by identical (monozygotic; MZ) twin pairs [36]. Twin heritability studies as well as the assumption that twins raised together experience similar environmental conditions [37]. Using 416 twin pairs and more than 1000 fecal samples from the TwinsUK community, Goodrich and colleagues in 2014 reported numerous microbial taxa whose abundances were impacted by host genetics [35]. Particularly, the family Christensenellaceae (phylum Firmicutes) indicated the most heritable taxon found. Additionally, people with low body mass index (BMI) showed an enriched network of Christensenellaceae and its associates. Because of these findings, Goodrich and colleagues went further to modify obese-associated microbiota with *Christensenella minuta*, a cultivated member of the Christensenellaceae, and then implanted them into germ-free mice. The result revealed that the receiving mice’s microbiota was changed, and the *C. minuta* amendment decreased weight gain. Although previous studies with relatively smaller twin numbers, 54 [38] and 87 [29], indicated no meaningful difference in the composition of gut microbiota between MZ and DZ twins, the difference became statistically significant with the larger sample, 416 [35] and 1126 [39]. Furthermore, studies have confirmed the relationship between the gene for lactase (*LCT*) and the relative abundance of *Bifidobacterium* (phylum Actinobacteria) [40,41,42].

### 2.4. Geographical Location

The environment in which a person resides can play a crucial role in determining the structure and composition of a person’s gut microbiota. To examine how gut microbiomes vary between human populations, Yatsunenko and colleagues characterized the bacterial species and their gene content found in fecal samples taken from 531 healthy individuals representing Amerindians from the Venezuelan Amazon, people living in rural Malawi, and people living in urban areas in the United States [29]. The findings reveal that people living in the USA have significantly different bacterial species compositions and functional gene repertoires than people living in the other two nations, thus indicating the possible impact of Westernization on gut microbiota. Due to their shared genes, similar bacterial richness and heritability for MZ twins compared to DZ twins have been observed [35,39]. However, twins’ microbial similarity decreased when they started to live apart, suggesting that the environment may have a greater influence on the gut microbiota than does genetics [43]. Furthermore, a non-twin investigation [40] revealed a considerable bacterial species similarity among genetically unconnected people who lived together, but not among family members who did not live together. Family members that live together as well as with their pets share similar microbiota [44], indicating that the structure and composition of people’s microbiota may be profoundly shaped by their direct and regular contact with cohabitants. Additionally, traditional diets vary by location, which may have an impact on the gut microbiome’s makeup. People who live in rural regions and eat more fermented foods, for instance, might have a distinct gut flora than people who live in cities and eat more processed foods. Evidence indicated that fermented dairy products consumed by nomadic Bedouins presented significantly more bacterial species, characteristic to the gut microbiota, compared to urban Saudis [45]. One of the main causes of fast-disappearing biodiversity is expanding urbanization. Residents of rural areas are more exposed to environmental microorganisms than residents of metropolitan areas [46]. In a study conducted by Parajuli and colleagues, it was observed that the diversity of Actinobacterial, Proteobacterial, Firmicutes, and Bacteroidetes communities decrease with urbanization [47]. Evidence also showed that people who live in warmer regions have a distinct gut microbiota from people who live in colder areas [48].

### 2.5. Other Factors

Throughout the world, antibiotics are a treatment of choice for bacterial infections. However, the downside is that they can disrupt the balance of the gut microbiome. Antibiotic use can cause the microbiota to become less diverse and rid the gut of beneficial bacteria which have an impact on people’s health and how resistant they are to illnesses [49]. A person’s gut microbiota can also be impacted by a lack of access to sanitary facilities. People who reside in unsanitary settings have been reported to be more susceptible to infections and disruption of the gut flora [50]. Moreover, stress has been implicated in gut microbiota imbalance. Host stress hormones are advantageous for enteric bacterial infections. Hormones secreted during host stress, such as catecholamines, can impact host–bacterial interactions, bacterial pathogenicity, and susceptibility to infection [51]. Enteric pathogens use stress hormones as signaling molecules to modify their virulence genes [52]. As an individual ages, changes in microbiota are introduced. Older adults show less diverse and imbalanced gut microbiota including immune system disruption and disease vulnerability [53]. Additionally, many lifestyle choices like drinking, smoking, and lack of physical activity have been reported to disrupt gut microbiota balance [54,55]. Eating soil—such as clay, dirt, chalk, or other kinds of soil—is a behavior known as geophagia. In some countries and regions, it is a popular practice, yet it can be harmful to the gut microbiota [56]. Heavy metals, herbicides, and other polluting residues found in the soil can disrupt the gut microbiota and promote the growth of pathogens [57].

To perform activities, gut microbiota must communicate using a sophisticated cell–cell system known as quorum sensing (QS). Studies reveal that QS has a pivotal role in gut microbiota homeostasis [58,59,60]. This suggests that interference in QS could disrupt gut microbiota balance and lead to disease conditions. Recently, we demonstrated that artificial sweeteners exert inhibitory effects on bacterial QS and proposed that QS inhibition could be one mechanism behind dysbiosis [61,62]. In this review, we discuss the role of QS in normobiosis. Beginning from the discovery of QS, we highlight the discovery of the numerous signaling molecules utilized in QS. We also evaluate strategies that enhance gut bacterial activity through QS activation and provide possible future direction. 

## 3. Quorum Sensing

Our understanding of chemical communication among bacteria has undergone a significant shift since the discovery of QS. From being seen as distinct noncooperative species, bacteria are now understood as socially cooperative organisms with the ability to engage in multicellular behaviors [63]. Via QS, bacteria live a multicellular life, coordinating group behaviors that are often impossible for a single cell to carry out [64]. Hastings and colleagues [65] were the first to discover QS over 50 years ago. In their investigation, they found that *Vibrio fischeri* (known then as *Photobacterium fisheri*) a bioluminescent bacterium rapidly produced light, which was not a result of cell growth but rather the “conditioning” of media by the developing cells. In freshly inoculated cultures, the bacterial cells did not begin to produce light until the mid-logarithmic phase. The researchers later named this conditioning phenomenon “autoinduction”. To describe and make the phenomenon clearer, Greenberg and colleagues introduced the phrase “quorum sensing” in 1994 [66]. Later studies showed that QS exists across various bacterial species, including pathogens [67,68,69,70,71,72,73,74,75].

QS is driven by chemical messengers known as autoinducers (AI), by which bacteria communicate with one another and coordinate their gene expression in response to their population density [71,72,73]. In the gut though, not every species produces AIs. When bacteria cannot produce their own AIs, they secretly “listen” to the signals produced by other bacteria, a phenomenon known as “eavesdropping” [76]. Using QS, numerous group activities are performed. These activities include biofilm formation [77,78], virulence factor production [74,79,80], sporulation [81,82], bioluminescence [65,83], nucleotide biosynthesis [84], DNA horizontal transfer [81,85], antibiotic synthesis [86], glucose uptake [70,84,87], adaptation to noxious environments [88,89], and production of secondary metabolites [90]. Interestingly, QS can occur within species and between species [90,91], as well as between kingdoms (e.g., eucaryotic host cells and bacteria) [92,93,94,95,96]. Therefore, the term “quorum sensing” can be expanded to encompass multimodal communication networks including the intraspecies, interspecies, and interkingdom signaling cascades.

## 4. Different Quorum Sensing Signals in the Gut (Figure 1)

### 4.1. Autoinducing Peptides (See Table 1)

Different AIs are employed by Gram-positive and Gram-negative bacteria. The Gram-positive bacteria utilize oligopeptides, commonly referred to as autoinducing peptides (AIPs), for their communication [97]. These AIPs are of various types, differing in sequence and structural organization [97,98]. Following their synthesis, AIPs are immediately processed. Since peptides are unable to traverse lipid-bilayer membranes, specialized transporters move the processed AIPs outside the cell [64]. The AIPs’ processing by posttranslational modification produces a number of products with sizes ranging from 5 to 17 amino acids in linear or cyclical structural organization [97,98]. The extracellular AIPs bind to a two-component histidine kinase receptor on the bacterial membrane and activate the kinase activity of the receptor. This results in autophosphorylation and the subsequent relay of the phosphate group to a response regulator downstream [99,100]. The phosphorylated response regulator activates the operon, creating an autoinducing feed-forward looping that synchronizes the QS response [64]. Some Gram-positive bacteria lack membrane-bound receptors. Therefore, the AIPs are returned to the cells’ cytoplasm with the aid of a transporter where they interact with the transcription factors, forming a regulatory complex that modulates gene expression [98,101].

**Figure 1:**
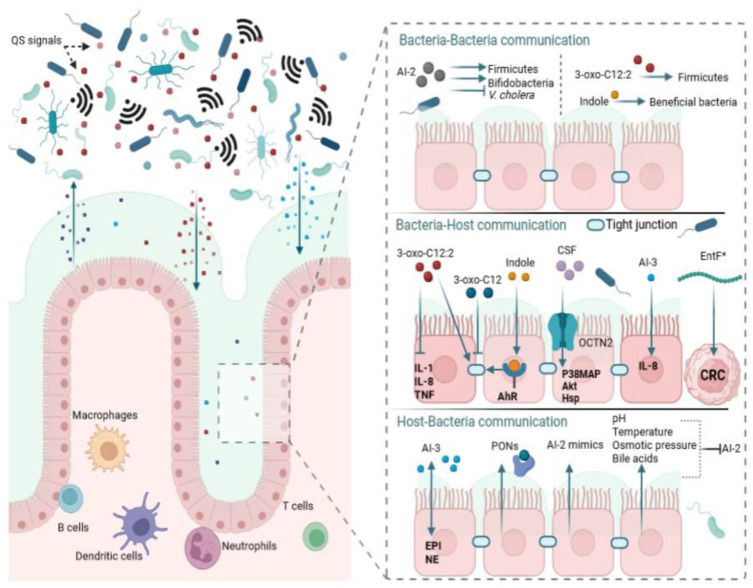
Cross-communication in the gut, showing bacteria–bacteria, bacteria–host, and host–bacteria communication. In the bacteria–bacteria communication, elevated AI-2 induces the growth of native gut bacterial residents such as Firmicutes and Bifidobacteria but suppresses the expansion of pathogens such as *V. cholera*. 3-Oxo-C12:2 is positively associated with Firmicutes. Indole enhances the proliferation of beneficial bacteria. In the bacteria–host communication, 3-oxo-C12:2 protect tight junction integrity. Indoles enhances epithelial barrier function via the aryl hydrocarbon receptor (AhR). While AI-3 induces pro-inflammatory reactions by stimulating the expression of cytokines IL-8, 3-oxo-C12:2-HSL reduces inflammation by repressing the expression of IL-1, IL-8, and TNF. 3-Oxo-C12 exacts a negative impact on the epithelial barrier by disrupting the tight junctions. Gram-positive signal peptides, such as competence and sporulation factor (CSF) bind to the cation transporter (OCTN2), and subsequently activate heat shock protein (HSP), p38 MAP, and protein kinase B (Akt), to protect intestinal barriers from oxidative stress damage, and impairment. The QS peptide, EntF*, promotes colorectal cancer (CRC) metastasis through interference with the epithelial cells’ integrity. The host–bacteria communication means, from the host end, the microenvironment in the lumen (pH, Temperature, Osmotic pressure, and Bile acids) which inactivates QS signals such as AI-2 is modulated. Also, paraoxonases (PONs) exert lactonase-like activity against AHLs signals. AI-2 mimics, and catecholamines (epinephrine (EPI)/norepinephrine (NE)) are recognized by the bacterial QS receptors to modulate gut microbial balance (created using BioRender.com, accessed on 12 December 2022).

**Table 1:** Quorum sensing molecules in the gut.

Quorum Sensing Molecules	Source	Structure	References
**Autoinducing peptides**	Gram-positive bacteria 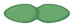	Oligopeptides with amino acids residues ranging from 5–17 arrange linearly or cyclical	[97,98]
***N*-acyl homoserine lactones**	Gram-negative bacteria 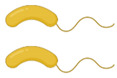	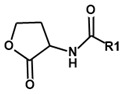 Homoserine lactone moiety with different acyl chain lengths or substitutions (R1)	[102,103]
**Autoinducer-2**	Both Gram-positive and Gram-negative bacteria 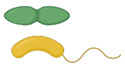	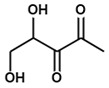 4,5-dihydroxy-2,3-pentanedione (DHPD), spontaneously cyclize and rearrange into AI-2	[68,104,105]
**Autoinducer-3**	Enteropathogenic *Escherichia coli* 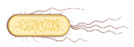	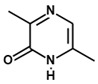 Pyrazinone family	[79,106,107]
**Indole**	Some Gram-positive and Gram-negative bacteria 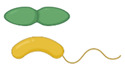	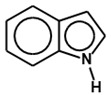 Produced by >100 species of Gram-positive and Gram-negative bacteria excluding *P. aeruginosa*, *A. niger*, and *S. enteric*	[108]
**Alkyl quinolones**	Some Gram-negative bacteria 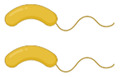	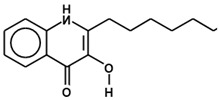 Alkyl quinolone derivative, known as *Pseudomonas* quinolone signal (PQS)	[109,110]
**Diffusible signal factor**	Some Gram-negative bacteria 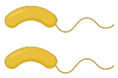	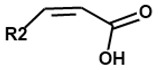 *Cis*-2-unsaturated fatty acids of various chain lengths and branching (R2)	[111,112,113,114]

### 4.2. N-Acyl Homoserine Lactones (See Table 1)

In Gram-negative bacteria, the most prevalent group of AIs are the *N*-acyl homoserine lactones (AHLs) [71,115]. Different bacterial species produce AHLs with different acyl chain lengths or substitutions [102,103], but with the same essential homoserine lactone moiety (Table 1). The LuxI protein family is responsible for producing the AHLs from *S*-adenosyl methionine (SAM) and an acylated-acyl carrier protein (ACP) [116]. The AHLs can freely move across the cell membrane [75,80]. At an extracellular threshold concentration, the AIs enter the cell and once in the cytoplasm and bound to their receptors, form a regulatory complex that activates the transcription of QS-control genes. Some Gram-negative bacteria have two-component histidine kinase receptors that function similarly to the Gram-positive two-component QS system [64,100,103,117]. The N-terminal domain of the QS regulatory protein LuxR (or its analog in other Gram-negative bacterial species) interacts with AHL, whereas the C-terminal domain interacts with DNA [118]. 

### 4.3. Autoinducer-2 (See Table 1)

Before the discovery of autoinducer-2 (AI-2), QS was thought to occur just within species employing AIP or AHL (collectively known as autoinducer-1 or AI-1). However, we now know that bacteria in mixed populations have different mechanisms by which they can sense, recognize, and interact with one another [68,104,105]. AI-2 is the “universal” QS molecule that mediates interspecies communication and was first identified in the marine bacterium *Vibrio harveyi* which, together with AHL, regulates bioluminescence [104,105]. Produced by *S*-ribosylhomocysteinase (LuxS), AI-2 is conserved in both Gram-positive and Gram-negative bacteria [91,119,120,121]. Using *S*-adenosylmethionine (SAM), bacterial cells produce *S*-adenosylhomocysteine (SAH) which is then broken down by the nucleosidase Pfs into adenine and *S*-ribosylhomocysteine (SRH) [122,123]. LuxS cleaves SRH at the thioether bond to produce homocysteine and 4,5-dihydroxy-2,3-pentanedione (DHPD), the latter of which cyclizes and rearranges spontaneously into AI-2 [68]. For bacteria, the step catalyzed by LuxS has at least two uses: SAH detoxification and AI-2 production [124].

### 4.4. Autoinducer-3 (See Table 1)

Autoinducer-3 (AI-3) consists of various products belonging to the pyrazinone family (Table 1) synthesize through a chain of reactions. Two of these reactions—the production of the AI-3 by the action of threonine dehydrogenase (Tdh) and the spontaneous cyclization by aminoacyl-tRNA synthetase—are crucial [79]. Studies have shown that the production of AI-3 is not dependent on AI-2 synthase (LuxS) [106,107]. AI-3 is responsible for the pathogenesis of enteropathogenic *Escherichia coli* (EPEC) [79,106,107,125,126]. QseC, a histidine kinase receptor, senses AI-3 to modulate gene expression [127]. QseC is conserved among different kinds of bacteria. Bacterial species such as *E. coli*, *V. cholerae*, *Salmonella* sp., *Shigella* sp., and *C. violaceium* possess QseC sensors [127,128]. Since bacterial QseC can also respond to host hormones (catecholamines), Kim et al. explored whether the AI-3 analogs may modulate human adrenergic signaling [79]. Their findings showed that AI-3 analogs have no impact on human adrenergic receptors. QseC lacks the primary sequence homology of adrenergic receptors [127]. 

### 4.5. Others (See Table 1)

Besides AI-2 and AI-3, indole is another signaling molecule that both Gram-positive and Gram-negative bacteria share (Table 1). Indole is produced by about 100 species of Gram-positive and Gram-negative bacteria, suggesting that it may also serve as an interspecies signal, although some bacterial species, such as *Pseudomonas aeruginosa*, *Aspergillus niger*, and *Salmonella enteric*, do not synthesize it [108]. Tryptophanase, an enzyme encoded by the *tnaA* gene, primarily found in commensal bacteria like *Bacteroides* spp. and *Clostridium* spp., breaks down tryptophan to produce indole [129]. Studies have shown that AI-2 and indole signaling are intertwined [108,130]. 

Other important QS molecules include alkyl quinolones (AQs). Some Gram-negative bacteria, such as *Burkholderia* and *Pseudomonas* genera, utilize the AQ-mediated QS system [109,110] besides the AHL-mediated QS system that is used by the majority of Gram-negative bacteria [71,72]. Although certain AQs, containing remarkable antibiotic properties, were first discovered in the 1940s in *P. aeruginosa*, it has only recently been found that the opportunist pathogen also produces an AQ derivative called the *Pseudomonas* quinolone signal (PQS) which is essential for bacterial communication [110]. Another QS family of molecules found in several Gram-negative bacterial species are the *cis*-2-unsaturated fatty acids, also known as the diffusible signal factors (DSFs) [114]. The first discovered DSF is *cis*-11-methyl-2-dodecenoic acid, which regulates the pathogenicity of the plant pathogen *Xanthomonas campestris pv campestris (Xcc)* [111,112]. Although DSF production was previously believed to be exclusive to xanthomonads, it is now recognized that other unrelated bacteria such as *Pseudomonas aeruginosa* and *Burkholderia cenocepacia* synthesize structurally similar molecules [113,114].

## 5. Role of QS in Normobiosis

### 5.1. Intraspecies QS and Normobiosis

Gut bacteria were initially thought to be eavesdropping on AHLs synthesized within the complex gut microbial ecosystem [131]. However, available evidence, recently obtained using highly sensitive and sophisticated technologies, indicates the existence of AHL signaling among native gut residents [58,59,132,133]. The discovery of AHL signaling in the gut which is linked to normobiosis is one of the significant findings in understanding the human gut. In the human fecal samples from patients with inflammatory bowel disease (IBD) and healthy individuals, 14 distinct AHLs were profiled, one of which was prominent, identified as 3-oxo-C12:2-HSL [93]. Evidence further showed that the patients with IBD had considerably lower levels of 3-oxo-C12:2-HSL molecules (16%) than individuals in good health (65.4%) [93]. The dramatic decrease in the 3-oxo-C12:2-HSL molecule in IBD patients was found to correspond with a gut microbiota imbalance characterized by a considerable decrease in important gut bacterial phyla including Firmicutes [58,134]. Firmicutes, together with Bacteroidetes, make up more than 90% of the gut microbial species in healthy individuals [3,135]. These prominent taxonomical groups perform a variety of tasks crucial to host health, including fermentation of a variety of complex polysaccharides to produce short-chain fatty acids (SCFAs) such as acetate, propionate, and butyrate [136,137]. SCFAs offer a variety of physiological benefits for the host such as tumor prevention [138], immunomodulation [139], and glucose and lipid regulation [140]. Firmicutes and Bacteroidetes species are believed to contribute differently to the pools of each SCFA. For example, the main butyrate producers are thought to be Firmicutes [141], whereas higher amounts of propionate and acetate in the total SCFA pool have been linked to the higher prevalence of Bacteroidetes [137,142]. For this reason, variations in the populations of these vital bacteria could affect the concentration of SCFAs, which in turn would have an impact on the host’s health. Indeed, reduced SCFAs have been observed in IBD [139,143,144]. 

### 5.2. Interspecies QS and Normobiosis

A growing body of knowledge shows that interspecies interactions mediated by Al-2 play a vital role in gut bacterial composition and balance. By using engineered *E. coli* strains that manipulate AI-2 levels by either raising or lowering the concentration of AI-2 in the gut, Thompson et al. evaluated the effect of the QS molecule on gut microbiota equilibrium [145]. In their studies, the researchers found that the antibiotic treatment lowered Firmicutes and other members carrying the *luxS* gene, indicating that AI-2 levels were reduced. Bacteroidetes were shown to dominate the microbiota following antibiotic-induced dysbiosis, possibly due to competitive advantage acquired from resistance caused by spontaneous mutations [146]. The streptomycin-induced dysbiosis from Thompson and colleagues’ study is consistent with those observed in other studies [147,148,149]. By elevating AI-2 levels, Thompson et al. observed that the growth of Firmicutes and other AI-2 producers was stimulated, whereas the proliferation of Bacteroidetes was suppressed, thus reversing the antibiotic-induced dysbiosis. More recently, in a study involving human patients and murine models of neonatal necrotizing enterocolitis (NEC), it was discovered that the concentration of AI-2 was dramatically reduced during the acute phase of the disease but gradually increased throughout the convalescent phase [150]. These findings show that AI-2 impacts the composition and equilibrium of gut microbiota. The link between AI-2 levels and intestinal microbiota balance suggests that the signaling molecules might be a novel biomarker for gut homeostasis.

Gut microbiota functions also include the suppression of pathogen expansion, a phenomenon known as colonization resistance. Direct interactions between gut microbial communities appear to be crucial in colonization resistance. In a study using fecal microbiota of adults living in a region with a high cholera burden, Hsiao et al. showed that the production of AI-2 by commensal bacteria conferred colonization resistance against *Vibrio cholerae* infection [151]. Among other commensal bacteria, *Ruminococcus obeum* was particularly prevalent in fecal samples during the recovery stage following *V. cholerae* infection. To elucidate the mechanism, Hsiao et al. artificially implanted gnotobiotic mice with a population of bacterial species from the human gut. Again, *R. obeum*, one of the species, significantly increased and prevented the colonization of *V. cholerae* through the expression of *luxS* (the gene for AI-2 synthase). Earlier, Bifidobacteriae were reported to offer protection against enterohemorrhagic *E. coli* (EHEC) [152] and *Citrobacter rodentium* infections [153] through colonization resistance. Studies have shown that the presence of AI-2 impacts Bifidobacteriae to form more biofilms, in turn promoting the colonization of the species [154,155]. It can be inferred, therefore, that AI-2 in the gut at least enhances the colonization fitness of the gut native bacterial community by promoting biofilm formation. When AI-2 activity was decreased using d-ribose, the biofilm formation of bacteriocin-producing *Lactobacillus paraplantarum* was inhibited [156]. AI-2 was shown to facilitate the auto-aggregation of *E. coli*, which promoted biofilm formation as well as enhanced bacterial stress resistance [157]. Taken together, these findings reveal that QS may have a role in suppressing pathogen expansion and restoring gut microbiota balance through enhancing biofilm formation and stress resistance in native gut bacteria. 

One of the crucial interspecies QS molecules produced by some bacteria is indole, an amino acid-derived metabolite [108,130]. At indole concentrations below 1 mM, *E. coli* was reported to only exhibit a repellent response, but switched to an attraction response when the indole concentration was at 1 mM or more [158]. Yang et al. suggest that indole may prevent pathogen invasion with a repulsion mechanism while bringing beneficial resident bacterial species together and enhancing their proliferation [158]. Through differential adaptation, gut bacteria in the presence of indole can suppress pathogen expansion. Enteropathogenic *E. coli* (EPEC) motility, epithelial cell adhesion, biofilm formation, and virulence gene expression were all reported to be reduced in the presence of indole [159,160,161]. Usually, co-infections with two or more infectious agents, such as *V. cholerae* and EPEC, have been observed in diarrheal samples [162]. Particularly, EPEC virulence has been demonstrated to increase in the presence of *V. cholerae*, possibly as a consequence of increased concentrations of *V. cholerae* AI-1 (CAI-1), the main QS molecule produced by *V. cholerae* [163]. Interestingly, the production of CAI-1 is inhibited by indole [163]. Additionally, the EPEC type III secretion system (T3SS), which was reported to be upregulated by the presence of CAI-1 [163], was inhibited by indole [164]. To infect host cells, EPEC uses the T3SS [165]. The interference of EPEC T3SS in the presence of indole was proposed to be due to the disruptive activity of indole to the CAI-1-mediated communication that enables EPEC to interact with *V. cholerae* [164]. This, therefore, suggests that the native resident bacteria of the gut could interfere with the pathogens’ virulence by thwarting their communication. By producing and rereleasing essential metabolites such as indole, native gut bacteria promote the fitness of their members as well as undermine the communication of enemies. 

### 5.3. Interkingdom QS and Normobiosis

The most documented inter-kingdom communications (host–bacteria interactions) are the ones driven by AHL molecules from pathogens [166]. Hosts adjust accordingly by monitoring AHLs within the gut ecosystem and resisting infection by interfering with QS signal transduction [92]. Enteric bacterial pathogens use host stress hormones to their advantage. Although the exact mechanisms behind microbiota and hormonal signaling are as yet unknown, hormones released during host stress can affect the host–bacteria interactions, bacterial pathogenicity, and vulnerability to infection [51]. These stress hormones may be exploited by enteric pathogens as signaling molecules to modulate their virulence genes [52]. The growth and motility of pathogenic bacteria like *Helicobacter pylori* [167], *Vibrio* spp. [128,168], *Klebsiella pneumoniae*, *P. aeruginosa*, *E. coli*, and *Staphylococcus aureus* [169] have been found to be modulated by catecholamines. Catecholamines have also been reported to increase bacterial virulence [170]. In *P. aeruginosa PA14*, virulence appears to be induced via the *las* QS pathway following norepinephrine treatment [171]. 

One of the mechanisms developed by the host in resisting infection is through influencing the activity of QS molecules. By modulating the gut physico–chemical environment including pH, temperature, osmotic pressure, bile acid levels, etc. the host may impact the functions of QS molecules such as AI-2 and thus influence gut microbiota activities (Figure 1) [172]. Besides regulating the gut environment, special QS-quenching enzymes are produced by host cells to modulate gut microbiota equilibrium. Enzymes such as PONsare known to degrade AHLs [173,174]. Three types of PON, which are all substantially conserved across species, have been identified (PON1, PON2, and PON3) [175] and are reported to be expressed in the gastrointestinal tract (GIT) [176]. Although PON1 and PON3 are primarily expressed in the liver and found in serum bound to high-density lipoprotein [177], PON2 is widely expressed and found in almost all human tissues including in the GIT but not detected in serum [178]. Higher levels of PON2 are reported in the jejunum than in the colon, ileum, and duodenum [179]. PON2 appears to have higher lactonase activity than the other two PON types [180,181]. PON2 deficiency in epithelia increased *P. aeruginosa* 3-oxo-C12-HSL-mediated QS, whereas the absence of PON1 or PON3 activity in epithelial cells did not affect the 3-oxo-C12-HSL [180]. Therefore, PON2 may be a key player in mammals’ inactivation of 3-oxo-C12-HSL [181]. The 3-oxo-C12-HSL from *P. aeruginosa* has been shown to have a negative impact on the epithelial barrier by disrupting the tight junctions, increasing paracellular permeability of macromolecules and ions [93,96]. The spread of pathogens such as *P. aeruginosa* with its associated effects could be mitigated by PON2. Although PONs’ primary physiological function and natural substrates remain largely unknown, it appears that they have roles in GIT pathologies. Patients with IBD express PON1 and PON3 at reduced levels when compared with healthy individuals [182]. On the other hand, carrying the PON1 R192 allele conferred protection against the onset of IBD in a case–control study conducted on Ashkenazi Jews [183].

Another important finding is the release of AI-2 mimics by human epithelial cells that are recognized by bacterial AI-2 receptors [95]. Although the mechanism of AI-2 mimic synthesis is still poorly understood, the data currently available indicate that AI-2 mimic activity is stimulated when epithelial cells are exposed to bacteria, either directly or indirectly, suggesting that one or more secreted bacterial components induce AI-2 mimic synthesis [95]. One can compare pathogen interactions with their host as an “arms race” in which each player continuously responds to the other’s changing tactics [92]. The human aryl hydrocarbon receptor (AhR), a protein well-known for its function in mediating toxicity [184], has been shown to interact with several QS molecules (such as 3-oxo-C12-HSL, C4-HSL, and PQS) produced by *P. aeruginosa* to keep track of the bacterial infection at various stages [92]. Such eavesdropping helps the host adapt to changes in the gut flora.

The suggestion that AHL molecules play a positive role in gut ecosystems is supported by 3-oxo-C12:2-HSL’s anti-inflammatory properties as well as its protective benefits on tight junction integrity (Figure 1) [93]. The chemokine IL-8, which is an important protein secreted by intestinal epithelial cells during inflammation, induces neutrophil recruitment in the mucosa and takes part in the acute-phase response [185,186]. Comparing the effects of 3-oxo-C12:2-HSL and the structurally similar AHL molecule 3-oxo-C12-HSL synthesized by *P. aeruginosa*, researchers found out that 3-oxo-C12:2-HSL, but not 3-oxo-C12-HSL, suppressed the stimulation of IL-8 secretion [93]. IL-8, induced by the pro-inflammatory cytokine IL-1 [187], was recently demonstrated to be downregulated, together with tumor necrosis factor (TNF), by 3-oxo-C12:2-HSL via bitter taste receptors [188]. While AI-3 could induce inflammatory reactions in a similar manner to other pyrazinones [79] through the expression of IL-8 [20,96], 3-oxo-C12:2-HSL [93] and AI-2 [189] were reported to reduce inflammation.

By stimulating beneficial bacteria and suppressing pathogenic bacteria in the intestines, indoles have an impact on the epithelial barrier [129]. Indole also has a direct impact on the epithelial barrier (Figure 1), exerting anti-inflammatory effects and immune system response by interacting with AhR [129]. The action of indoles on the AhR results in the induction of IL-22 [190] which enhances stem cell-mediated epithelial barrier repair and safeguards against infection and damage induced by hyperinflammatory reactions [191]. Furthermore, intestinal microbiota-produced indole metabolites were reported to reduce intestinal inflammation through activating type I interferon (IFN-1) signaling [192].

Compared to other QS signaling molecules, much less research has been done on the impact of AIP on gut microbiota balance. However, available data suggest the importance of host–bacteria interaction via AIP in gut microbial balance (Figure 1). An AIP from *Bacillus subtilis*, also known as competence and sporulation factor (CSF), was shown to contribute to normobiosis by mediating inter-kingdom signaling [193]. *B. subtilis* was previously thought to be just soil bacteria, however, it is now confirmed to be a member of the human gut and evolved to exist there [194,195]. Today, *Bacillus* species are commonly used as probiotics [196]. *B. subtilisis* administration was demonstrated in murine models to reduce dextrose sulfate sodium (DSS)-induced inflammation and dysbiosis by balancing the gut microbiota as well as related metabolites, with the resultant healing of intestinal mucosa damage impacted following DSS exposure [197]. The organic cation/carnitine transporter 2 (OCTN2), a cell membrane transporter, appears to be the channel through which CSF interacts with the host [193]. A significant correlation exists between mutations in the gene that codes for OCTN2 and susceptibility to Crohn’s disease [198,199]. CSF appears to reduce oxidative stress that causes cell death and breakdown of the epithelial barrier [193]. After being absorbed by intestinal epithelial cells via OCTN2, CSF triggers vital survival pathways such as p38 MAP kinase and protein kinase B (Akt) and stimulates cytoprotective heat shock proteins (HSPs), the latter of which protects gut epithelial cells from oxidative stress damage and impairment of barrier function [193]. These findings suggest that the interaction between QS molecules and host cells may make it easier for the host to adjust/adapt to changes in the microbiota that favors restoration and recovery of microbial equilibrium. Available evidence so far indicates that the function of OCTN2 may be limited to CSF [193]. This, therefore, suggests that other similar transporters such as OCTN1 [200] and MDR 1 (also known as P-glycoprotein or P-gp 1) [201] could complement OCTN2 in the surveillance of the gut microbiota by the host. Together, these transporters represent crucial systems for homeostatic host–bacteria interactions. Perhaps this system of host surveillance of gut ecosystem is one mechanism by which the host assesses alterations that might otherwise tip the balance of the gut microbiota. With the growing interest in AIPs, important discoveries are constantly being made. For example, a recently published study demonstrates that a new family of QS peptides, such as EntF*, produced by *Enterococcus faecium*, mediates host interaction and enhances colorectal cancer (CRC) metastasis [202]. In their findings, Wynendaele et al. showed that EntF* modulates the epithelial–mesenchymal transition (EMT) by regulating the expression of E-cadherin via the CXCR4 receptor [202]. Mechanistically, EMT enables solid tumors to develop into more malignant phenotypes and increases their invasive and metastatic properties [203]. 

## 6. Future Direction

Imbalance in the enteric bacterial community by a variety of agents can result in disease conditions [204,205,206,207]. Accordingly, restoring, or re-establishing, normobiosis may hold promise to address or even avoid dysbiosis-associated pathologies. Despite our knowledge of numerous receptor proteins that enable the host’s surveillance of the gut ecosystem for QS signaling molecules [184,193], the intracellular mammalian receptors or targets for such QS signals and the specific QS signaling pathways have not been elucidated yet. 

**Figure 2:**
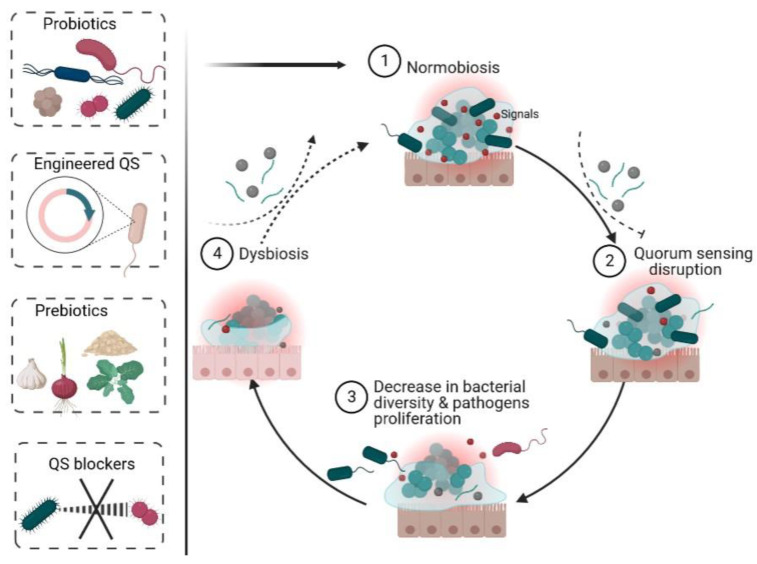
Opportunities for therapeutic interventions in restoring or re-establishing gut microbiota balance after dysbiosis. Normobiosis (1) can be altered through quorum sensing disruption (2). In turn, the quorum sensing disruption leads to decrease in bacterial diversity and pathogens proliferation (3), which eventually results in dysbiosis (4). Dysbiosis can be reversed using probiotics, prebiotics, engineered QS systems, or blockers of pathogens’ QS.

Understanding how enteric bacteria compete and cooperate is crucial for uncovering the processes of disorders associated with dysbiosis [208]. A recent report shows that targeting the LuxR-type receptors for bacterial QS may be a novel strategy for modulating the gut microbiota in IBD [134]. Quorum quenching, or the use of inhibitory chemicals (QS blockers) or enzymes, may offer promise for treating diseases (Figure 2). However, they may also possess the potential to inhibit the action of beneficial bacteria. Therefore, to develop effective therapeutic interventions, it is necessary to ensure that such therapies are target-specific, and not broad in their actions. This emphasizes the need for more knowledge about how QS is regulated and how various QS systems interact in the gut environment. With this understanding, we may be able to regulate bacterial community structure and functions to improve human health while also producing much-needed alternatives to antibiotics. The ease with which peptides may be synthesized and the revelation that some peptide-based QS molecules, such as CSF, function within a physiological concentration range (10–100 nM) [209] makes them promising candidates for therapeutic applications in restoring and reestablishing normobiosis.

On one hand, one could also think of QS molecules as trustworthy biomarkers for chronic dysbiosis-related disorders. The concentration of AI-2 is said to rise as adenomas develop into CRC [210]. On the other hand, in IBD, 3-oxo-C12:2-HSL decreases significantly [93]. To date, the source of 3-oxo-C12:2-HSL has not yet been identified. Although the 3-oxo-C12:2-HSL is strongly associated with Firmicutes, for the time being, it might be more appropriate to think of it as a marker of gut microbial balance as opposed to anything Firmicutes themselves synthesize [93]. Uncovering which bacteria synthesize 3-oxo-C12:2-HSL will advance our understanding and discussion on the role of AHLs in normobiosis. Given its protective function on intestinal epithelia and its loss in IBD patients [93,132,211], 3-oxo-C12:2-HSL could be targeted to reduce inflammation and possibly IBD. Furthermore, since the presence of AI-2 increases Firmicute count [145], hope is raised for therapeutic applications. The AI-2 molecule might be a useful intervention for redressing the balance between the prominent taxonomical groups. As demonstrated previously [145], recombinant AI-2-manipulating strains can be used as probiotics to restore substance-induced dysbiosis or suppress pathogen expansion. While the human epithelial cells release AI-2 mimics that are recognized by bacterial AI-2 receptors [95], a lot is still not understood about the process through which AI-2 mimics are synthesized. One can imagine a variety of approaches using AI-2 or mimics supplementation as QS-type prebiotics (Figure 2). Additionally, including organic, traditional, and personalized functional foods in the diet could help the body deal with numerous harmful stressors and the lifetime developmental epigenetic program, preventing genetic and epigenetic anomalies in the human metagenome [21]. 

Other microbial components of the gut, such as fungi, also show evidence of QS utilization besides bacteria [212,213]. Available data from other complex microbial ecosystems, like the soil, indicates the importance of QS in fungal activities [214]. Despite being less prevalent in the gut compared to bacteria, fungi are crucial to maintaining the equilibrium of this intricate gut microbial community [215,216]. Since our focus in this review is on bacterial QS, we intend, in a separate paper in the future, to discuss the QS signaling molecules produced by gut fungi and elucidate the mechanisms by which they influence bacteria and the host, including their potential for healthcare applications.

## 7. Conclusions

Since Hastings and colleagues discovered QS in 1970, our understanding of bacterial communication has improved substantially. Today, QS is broadened to include multimodal communication, encompassing intraspecies, interspecies, and interkingdom signaling. The role of QS in normobiosis is undeniable. Via QS, gut microbiota maintains balance by suppressing pathogen expansion through enhancing biofilm formation and fitness of resident gut bacteria, mobilizing native members to reestablish balance following substance-induced dysbiosis, exerting anti-inflammatory response as well as preserving the tight junction integrity. Although gut microbiota research has just scratched the surface, exciting prospects exist for QS-based therapeutic interventions. With the advancement in technology, more and more tools are made available to further clarify the role of QS in normobiosis and elucidate the connection between QS inhibition and dysbiosis. 

## Data Availability

Not applicable.
